# Scaling up and scaling down the production of galactaric acid from pectin using *Trichoderma reesei*

**DOI:** 10.1186/s12934-017-0736-3

**Published:** 2017-07-11

**Authors:** Toni Paasikallio, Anne Huuskonen, Marilyn G. Wiebe

**Affiliations:** VTT Technical Research Centre of Finland Ltd., P.O. Box 1000, 02044 Espoo, Finland

**Keywords:** Galactaric acid, Mucic acid, d-Galacturonic acid, Pectin, *Trichoderma reesei*, Scale-up, Scale-down

## Abstract

**Background:**

Bioconversion of d-galacturonic acid to galactaric (mucic) acid has previously been carried out in small scale (50–1000 mL) cultures, which produce tens of grams of galactaric acid. To obtain larger amounts of biologically produced galactaric acid, the process needed to be scaled up using a readily available technical substrate. Food grade pectin was selected as a readily available source of d-galacturonic acid for conversion to galactaric acid.

**Results:**

We demonstrated that the process using *Trichoderma reesei* QM6a Δ*gar1 udh* can be scaled up from 1 L to 10 and 250 L, replacing pure d-galacturonic acid with commercially available pectin. *T. reesei* produced 18 g L^−1^ galactaric acid from food-grade pectin (yield 1.00 g [g d-galacturonate consumed]^−1^) when grown at 1 L scale, 21 g L^−1^ galactaric acid (yield 1.11 g [g d-galacturonate consumed]^−1^) when grown at 10 L scale and 14 g L^−1^ galactaric acid (yield 0.77 g [g d-galacturonate consumed]^−1^) when grown at 250 L scale. Initial production rates were similar to those observed in 500 mL cultures with pure d-galacturonate as substrate. Approximately 2.8 kg galactaric acid was precipitated from the 250 L culture, representing a recovery of 77% of the galactaric acid in the supernatant. In addition to scaling up, we also demonstrated that the process could be scaled down to 4 mL for screening of production strains in 24-well plate format. Production of galactaric acid from pectin was assessed for three strains expressing uronate dehydrogenase under alternative promoters and up to 11 g L^−1^ galactaric acid were produced in the batch process.

**Conclusions:**

The process of producing galactaric acid by bioconversion with *T. reesei* was demonstrated to be equally efficient using pectin as it was with d-galacturonic acid. The 24-well plate batch process will be useful screening new constructs, but cannot replace process optimisation in bioreactors. Scaling up to 250 L demonstrated good reproducibility with the smaller scale but there was a loss in yield at 250 L which indicated that total biomass extraction and more efficient DSP would both be needed for a large scale process.

## Background

There is considerable interest in replacing chemicals derived from petroleum with bio-derived chemicals, i.e. chemicals obtained by bioconversion and/or chemical conversion from renewable biological resources, primarily plants. Both galactaric and glucaric acids have been identified as substrates for chemical conversion to adipic acid, furandicarboxylic acid (which is being developed as a substitute for terephthalic acid) and other platform chemicals, including anhydrides, diesters and diallyls which can be used in the synthesis of higher value products [1–3]. Both compounds can be prepared by nitric acid oxidation of the corresponding monosaccharide (d-glucose or d-galactose) [4]. Although biotechnological conversions may be preferable to nitric acid oxidation, biotechnological conversion of d-glucose to glucaric acid continues to be challenging [5]. However, galactaric acid has been produced from d-galacturonic acid using genetically modified *Escherichia coli* and *Trichoderma reesei* at concentrations of 10 [6] to 20 [7] g L^−1^, which are high enough concentrations for precipitation from the culture broth, making biotechnologically derived galactaric acid available for assessment in further chemical conversions [6]. The genetically modified *E. coli* or *T. reesei* convert d-galacturonic acid to galactaric acid by the action of a uronate dehydrogenase (UDH), which is expressed in a strain in which normal d-galacturonic acid metabolism has been disrupted [6, 8]. In the case of *T. reesei*, deletion of the gene encoding NADPH-dependent d-galacturonate reductase (*gar1*), the first step in the fungal pathway for d-galacturonic acid metabolism, is sufficient to disrupt the pathway and expression of the *A. tumefaciens udh* gene results in a strain which produces galactaric acid [8].

Although chemically produced galactaric acid is commercially available, biotechnologically produced galactaric acid will contain different impurities and the effect of these on specific chemical reactions needs to be assessed to provide feedback on the purity requirements and consequent implications for downstream processing (DSP) in the biotechnological process. For this reason it may be necessary to produce substantial quantities of galactaric acid using biotechnology, even though the process is not ready for commercialisation.

The substrate for biotechnological production of galactaric acid is d-galacturonic acid, which can be obtained by hydrolysis of pectin. Zhang et al. [6] demonstrated that galactaric acid could be produced from enzymatically hydrolysed sugar beet pulp, but noted that the yield of galactaric acid on sugar beet pulp was relatively low (0.14 g galactaric acid per g sugar beet pulp), reflecting the high concentration of other compounds in the pulp, particularly d-glucose and l-arabinose. d-Galacturonic acid is currently available only at high prices (more than €3000 per kg) and is thus not suitable for producing kilogram amounts of galactaric acid for chemical testing. However, food grade pectins are commercially available at prices in the range of €10–100 per kg, making them a reasonable source for preparation of large amounts of galactaric acid. It should be noted that food grade pectin may be diluted with additives such as sucrose or glucose. Having received a request to provide a 2 kg sample of biotechnologically produced galactaric acid for use in chemical reactions, we decided to use pectin as the source of d-galacturonic acid in scaling up the process of galactaric acid production with *T. reesei* Δ*gar1 udh*. An industrial process would be expected to use sugar beet pulp, citrus waste and other pectin-rich waste streams or crude extractions of pectin from these sources, which would not compete with the food use of current pectin production, but these were not readily available at the scale needed. *T. reesei* Δ*gar1 udh* was used in scaling up the process since it has already been demonstrated to produce up to 20 g L^−1^ galactaric acid from d-galacturonic acid [7]. Since *T. reesei* does not hydrolyse pectin [9] and to facilitate sterilisation of viscous pectin solutions, enzymatic pre-hydrolysis of the pectin was necessary. An alternative approach would be to develop a consolidated process using a production strain such as *Aspergillus niger*, which produces native pectinases. However, *A. niger* can metabolise galactaric acid [8] and a strain in which this pathway has been disrupted has only recently become available [10]. A production process with *A. niger* has not yet been developed.

In addition to scaling up the process of producing galactaric acid to provide galactaric acid for chemical reactions, we were also interested in providing a scaled down process, which would enable the screening of new strains [11]. Galactaric acid production by *T. reesei* has previously been demonstrated in flask cultures [8], but at much lower concentrations than can be obtained in bioreactors [7]. Running and Bansal [12] demonstrated that 24-well plates can have as good or better oxygen transfer as shaken flasks, depending on the shaking regime applied, and 24-well plates are increasingly being used for the cultivation of filamentous fungi [13–15]. It is therefore useful to assess whether galactaric acid production by *T. reesei* could be scaled down for production in 24-well plates using pectin as substrate.

In this paper we describe the production of galactaric acid by *T. reesei* VTT D-161646 from enzyme hydrolysed pectin at 1, 10 and 250 L scales. We also demonstrate that the process can be scaled down to 4 mL for strain screening, for example in considering the effectiveness of alternative promoters for expression of the uronate dehydrogenase gene, as shown here.

## Results

### Scaling down production of galactaric acid in 24-well plates

**Fig. 1:**
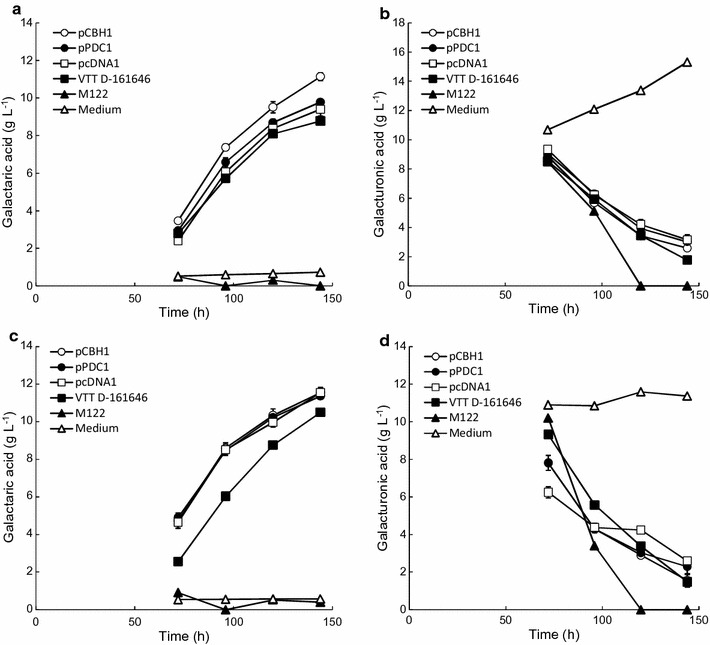
Galactaric acid production and d-galacturonic acid consumption in 24-well plates. *T. reesei* Δ*gar1 udh* strains, in which the *udh* gene was under control of the *CBH1*, *PDC1*, *cDNA1* or *GPDA* (VTT D-161646) promoters, and M122, which contains *gar1* and does not contain *udh*, were grown in 4 mL medium containing pure d-galacturonate (**a**, **b**) or hydrolysed pectin (**c**, **d**) as substrate in 24-well plates. All values have been adjusted for evaporation, except those for the medium in wells with pure d-galacturonic acid. Values for p*CBH1*, p*PDC1* and p*cDNA1* represent mean ± sem for n = 3. Where not seen, *error bars* were smaller than the *symbols*

Three transformants of *T. reesei* were generated in which the uronate dehydrogenase (*udh*) gene was expressed under different promoters and these were cultivated in 24-well plates to assess the suitability of the 24-well plate format for galactaric acid production. All transformants which contained the *udh* gene produced galactaric acid in the 24-well plates. *T. reesei* VTT D-161646 produced 8.8 g L^−1^ galactaric acid from 10.7 g d-galacturonic acid in 6 days and 10.5 g L^−1^ from 20 g L^−1^ pectin (Sigma), hydrolysed to give 11.6 g L^−1^ d-galacturonic acid, demonstrating that galactaric acid could be produced in the 24-well plates (Fig. 1; Table 1). No galactaric acid was produced by M122, the control strain lacking *udh*, nor in wells which had not been inoculated (Fig. 1). The increase in concentration of d-galacturonic acid (Fig. 1b) and lactose in the uninoculated wells provided evidence of the extent of evaporation in the wells and this data was used to calculate the actual concentrations of galactaric acid produced in other wells. Hydrolysis of the pectin was not complete within 72 h, so changes in the concentrations of d-galacturonic acid and lactose in the well with medium containing d-galacturonate were used to adjust for evaporation in the pectin containing wells.

**Table 1:** Galactaric acid production by *T. reesei* expressing *udh* under promoters p*CBH1*, p*cDNA1*, p*PDC1* or p*GPDA*

Strain	p*CBH1*	p*cDNA1*	p*PDC1*	p*GPDA* ^a^
24-well plates—d-galacturonate				
Galactaric acid (g L^−1^)	11.1 ± 0.2	9.4 ± 0.4	9.8 ± 0.1	8.8
Galactaric acid production rate (72–120 h) (g l^−1^ h^−1^)	0.13 ± 0.01	0.12 ± 0.00	0.12 ± 0.00	0.11
Yield galactaric/d-galacturonic (g g^−1^)	0.95 ± 0.02	0.87 ± 0.02	0.89 ± 0.00	0.89
24-well plates—pectin				
Galactaric acid (g L^−1^)	11.6 ± 0.2	11.5 ± 0.3	11.4 ± 0.1	10.5
Galactaric acid production rate (72–120 h) (g L^−1^ h^−1^)	0.12 ± 0.04	0.11 ± 0.00	0.11 ± 0.00	0.13
Yield galactaric/d-galacturonic (g g^−1^)	0.84 ± 0.03	0.90 ± 0.03	0.89 ± 0.03	0.76
0.5 L bioreactors—d-galacturonate				
Galactaric acid (g L^−1^)	15.7	13.6	16.0	19.7
Galactaric acid production rate (0–72 h) (g L^−1^ h^−1^)	0.22	0.21	0.09	0.19
Yield galactaric/d-galacturonic (g g^−1^)	0.90	0.82	1.11	0.85
Maximum biomass (g L^−1^)	9.5 ± 0.2	9.0 ± 0.3	6.5 ± 0.5	7.9 ± 0.3

Galactaric acid production (titre, rate of production and yield on d-galacturonic acid) in 24-well plates and in 0.5 L bioreactors using either d-galacturonic acid or pectin (Sigma) as substrate, with lactose as co-substrate. Maximum biomass in bioreactors was observed at 50 (p*CBH1*, p*cDNA1*, p*GPDA*) or 72 (p*PDC1*) h after which time biomass decreases (see also Fig. 3). Values are mean ± sem for three replicates

^a^Strain VTT D-161646

Each of the new transformants produced similar amounts of galactaric acid to VTT D-161646 and to each other (Fig. 1; Table 1). The strain expressing *udh* under control of the CBH1 (cellobiohydrolase I) promoter produced significantly more (p < 0.05) galactaric acid (11.2 ± 0.2 g L^−1^) from d-galacturonic acid than the other three strains, but there were no significant differences (p > 0.05) in the amounts produced from Sigma pectin (Fig. 1). The yield of galactaric acid on d-galacturonic acid was between 0.87 and 1.00 g g^−1^ in the 24-well plates when d-galacturonic acid was used as the substrate, and between 0.76 and 0.90 g g^−1^ when pectin was used as substrate (Table 1). Production rates between 72 and 120 h were 0.11–0.13 g L^−1^ h^−1^ with either pure d-galacturonic acid or with pectin as the source of d-galacturonic acid (Table 1).

In lactose-d-galacturonic acid fed-batch cultures (0.5 L, using the optimised process described by [7]) transformants expressing *udh* under the *gpdA* (glyceraldehyde-3-phosphate dehydrogenase, strain VTT D-161646), *CBH1*, *cDNA1* (unidentified hypothetical protein), and *PDC1* (pyruvate decarboxylase) promoters produced 20, 16, 14 and 16 g L^−1^ galactaric acid, respectively (Table 1). Yields were 0.85, 0.90, 0.82 and 1.11 g galactaric acid [g d-galacturonate consumed]^−1^, respectively. However, the strain producing galactaric acid under the *pdc1* promoter grew slower than the other strains, producing less biomass, and the initial production rate (between 0 and 72 h) of d-galactaric acid (0.09 g L^−1^ h^−1^) was slower than that of the other three strains (0.19–0.20 g L^−1^ h^−1^, Table 1).

### Production of galactaric acid from pectin at 1, 10 and 250 L scale

**Fig. 2:**
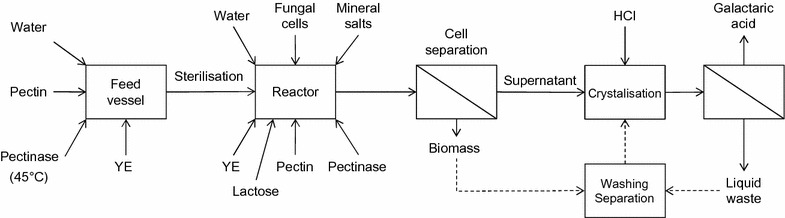
Process scheme for production of galactaric acid from pectin. Input concentrations are given in the methods and outputs are described in “Results”. Process steps carried out here are indicated with *solid lines*. *Broken lines* illustrate the potential for an additional biomass extraction step, with liquid recycling. Filtration was used for cell separation and for collection of galactaric acid crystals

Strain VTT D-161646 was used in the scaling up experiments since production conditions have been developed specifically for this strain [7] and there was no improvement in galactaric acid production with the new strains under these conditions. In order to scale up production of galactaric acid, d-galacturonic acid was replaced with pectin, using either Sigma or Meridianstar pectin (Table 2). A process scheme for the production of galactaric acid from pectin is shown in Fig. 2. The 10 L culture was carried out using Sigma pectin while waiting for the Meridianstar pectin to arrive. Because of the high d-glucose content in the Meridianstar pectin it was necessary to adjust the process at 1 L scale to assess the extent to which the high d-glucose content would repress galactaric acid production. Therefore both 1 and 250 L cultures were carried out using Meridianstar pectin.

**Table 2:** Carbohydrate composition (% dry matter) of pectin

Pectin	Sigma P1935	Meridianstar rapid set
d-Galacturonate	65.6	43.0
d-Glucose	1.7	35.5
l-Arabinose	1.2	1.5
d-Galactose + d-xylose	11	4

Pectin was hydrolysed by addition of 0.5–1.0 mL L^−1^ Pectinex Ultra with 0.1 mL L^−1^ Pectinex Smash and incubation at 40 °C. d-Galactose and d-xylose were not separated on the HPLC column used

**Fig. 3:**
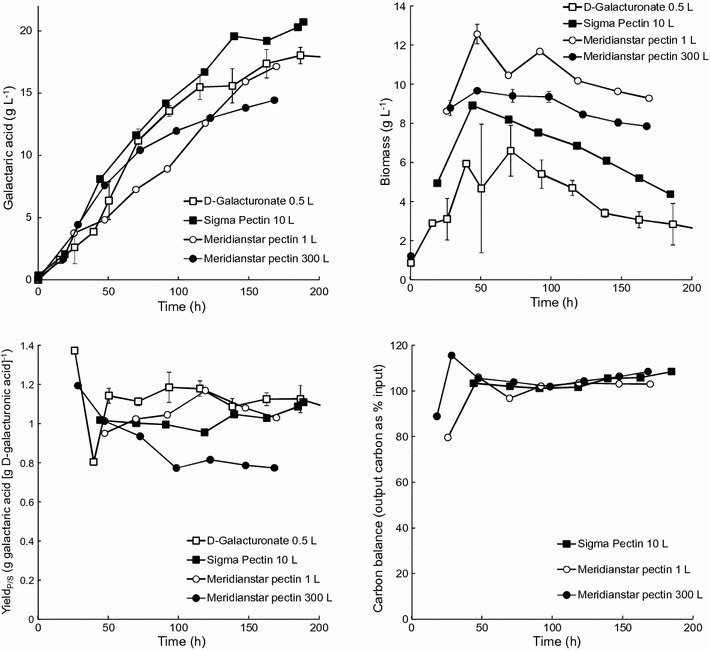
Galactaric acid and biomass production at 0.5, 1, 10 and 250 L scale. *T. reesei* Δ*gar1 udh* VTT D-161646 was provided d-galacturonic acid (0.5 L, [7]) or hydrolysed pectin (1, 10 and 250 L) as substrate with lactose as co-substrate. The approximate yield of galactaric acid on d-galacturonic acid and the overall carbon balance are also shown. Values for production from d-galacturonic acid at 0.5 L scale are mean ± sem for 4 independent cultivations. *Error bars* for biomass measurements at 1, 10 and 300 L scale are ±sem for duplicate measurements

When Sigma pectin replaced d-galacturonic acid in the *T. reesei* galactaric acid production process, 21 g L^−1^ galactaric acid was produced with a yield of 1.11 g galactaric acid [g d-galacturonic acid]^−1^, assuming that the pectin had been fully hydrolysed. The yield of galactaric acid on pectin was 0.73 g g^−1^. The production rate during the first 140 h (0.14 g L^−1^ h^−1^) was comparable to that observed previously with pure d-galacturonic acid during the same time interval (0.15 g L^−1^ h^−1^; Fig. 3) [7] and the process generally showed good reproducibility with the 500 mL scale production from d-galacturonic acid. No accumulation of d-galacturonic acid or other carbohydrates (lactose, glucose, galactose or arabinose) was observed during the feeding phase. Biomass production on pectin with lactose was a bit higher than on d-galacturonic acid with lactose, even though the lactose concentration had been reduced to take into account the carbohydrates introduced with the pectin (Fig. 3). d-Glucose remained limiting throughout the feeding phase.

The culture broth was collected at the end of the cultivation and the biomass separated by filtration, generating 8.8 L clear liquid and 282 g wet biomass. Precipitating and collecting the crystals of galactaric acid resulted in 154 g galactaric acid, representing 85% of the galactaric acid produced (Table 3).

**Table 3:** Process parameters for galactaric acid production at 0.5, 1, 10 and 250 L

	d-Galacturonic acid^a^	Meridianstar pectin	Sigma pectin	Meridianstar pectin
Reactor volume (L)	0.5	1	10	250
Process time (h)	242	215	185	168
Inputs				
Lactose (g L^−1^)	28	10	14	9
Galacturonic acid (g L^−1^)	21			
Pectin (g L^−1^)		48.4	29.2	45.9
Pectinase(s) (mL L^−1^)		1	1	2
Fungal cells (g L^−1^)	1	1	1	1
Air (vvm)	1.4	0.5	0.5	0.5
Output				
Galactaric acid (g L^−1^)	21 ± 2	18	21	15
Yield (g [g galacturonate]^−1^)	1.0 ± 0.2	1.0	1.1	0.8
Yield (g [g pectin]^−1^)		0.41	0.73	0.32
Biomass (g L^−1^)^b^	4 ± 1	9 ± 0.1	4 ± 0.1	8 ± 0.2
Product recovery (kg)		0.014	0.15	2.8
Product recovery (%)	68 ± 7	89	85	73

^a^Data from [7]. Values are mean ± sem for four cultivations

^b^Biomass measurements are mean ± sem for 2–3 replicates, except for 0.5 L cultivations (mean ± sem for 4 cultivations), at end of cultivation

Using Meridianstar Rapid Set pectin, 19 g L^−1^ galactaric acid was produced with a yield of 1.00 g galactaric acid [g d-galacturonic acid]^−1^, assuming complete hydrolysis of the pectin (Fig. 3). The yield of galactaric acid on Meridianstar pectin was 0.41 g g^−1^. Feeding was not started until all d-glucose and lactose had been consumed, but this resulted in d-galacturonic acid limitation during the batch phase and reduced the initial galactaric acid production rate. The initial production rate (0–70 h) was only 0.10 g L^−1^ h^−1^, but during the feeding phase (90–150 h) it increased to 0.13 g L^−1^ h^−1^. Although there was no accumulation of d-glucose during the feeding phase, there was initial accumulation of d-galacturonic acid (up to 4.3 g L^−1^) in the culture supernatant and the feed rate was reduced to allow more complete conversion to d-galactaric acid. More biomass was produced when *T. reesei* VTT D-161646 was grown on Meridianstar pectin than on Sigma pectin and the biomass concentration was maintained during the feeding phase (Fig. 3). Approximately 89% of the galactaric acid was recovered by precipitation in acid (Table 3).

Based on the results at the 1 L scale, *T. reesei* VTT D-161646 was grown in a 300 L bioreactor and 256 L of 14 g L^−1^ galactaric acid was produced. The initial (0–73 h) production rate of 0.15 g L^−1^ h^−1^ was only sustained for 72 h, after which time both the rate of galactaric acid production and the yield of galactaric acid on d-galacturonic acid decreased. The final yield was 0.77 g galactaric acid [g D-galacturonic acid]^−1^ or 0.32 g [g pectin]^−1^ (Fig. 3). Crystals were observed among the mycelia under the microscope and were also present in the foam which had been produced in the bioreactor. After precipitation, collection and drying, 2.81 kg galactaric acid was obtained, or 73% of the amount present in the supernatant (Table 3). Biomass was not extracted.

## Discussion

The process of producing galactaric acid using *T. reesei* was scalable both up and down, with little loss in productivity, even with the substitution of pectin for d-galacturonic acid. Pectin was found to be a suitable substrate for galactaric acid production, although for *T. reesei* this required the addition of commercial pectinases. Addition of pectinases also facilitated sterilisation of the pectin feed, so even though *T. reesei* could be engineered to express a full range of pectinases to reduce the cost of enzyme addition, a fully consolidated process may not be desirable. The only other adjustment required for the use of pectin was a reduction in the amount of added co-substrate, since co-substrates were provided with the pectin, particularly in the case of the food-grade pectin, which contained a high concentration of d-glucose. Earlier results had indicated that d-glucose-limited fed-batch culture was less effective than lactose-limited fed-batch culture for galactaric acid production with *T. reesei* VTT D-161646 [7], but this did not appear to be the case when d-glucose was supplied with hydrolysed pectin in the feed.

Pectin was also used in the 24-well plates, with no apparent problems with mixing. Evaporation could be a problem at this scale, however, as seen in the increasing concentration of substrates in uninoculated wells, and needed to be taken into account. Production from hydrolysed pectin was comparable to that observed when pure d-galacturonic acid was provided. Production of galactaric acid was much higher in these 4 mL batch cultures than has previously been reported for flask cultures with filamentous fungi (around 4 g L^−1^; [8, 10]). The medium in the 24-well plates contained spent grain extract, which was expected to induce the CBH1 promoter [16], but not necessarily other promoters. Spent grain extract also provides organic nitrogen, resulting in a richer medium, which may contribute to the good conversion of d-galacturonic acid into galactaric acid. Kuivanen et al. [10] recently found that providing yeast extract and peptone to a *A. niger* galactaric acid producing strain, resulted in improved galactaric acid production, although the amounts were still low.

Production of galactaric acid by three new strains was compared in the 24-well plates, but production of all strains was found to be similar. This suggested that the amount of uronate dehydrogenase produced under the control of each of the promoters tested (p*CBH1*, p*cDNA1*, p*PDC1* and the original p*GPDA*) was adequate to catalyse the conversion of d-galacturonic acid to galactaric acid at the rate at which it was taken up under these conditions. The effectiveness of these strains in producing galactaric acid was confirmed in 0.5 L bioreactors, however, the bioreactor cultures also revealed a slow growth (and production) phenotype for the strain expressing *udh* under the *PDC1* promoter, which was not observed in the microtiter plates. The *PDC* promoter is expected to be particularly well induced when d-glucose is the main carbon source, and conversion of d-galacturonic to galactaric acid may be better with d-glucose as co-substrate [17], rather than lactose, as used here. The provision of better aeration and substrate-limited feeding in the bioreactors may also contribute to the phenotype.

The yield of galactaric acid on d-galacturonate was close to the theoretical yield of 1.08 g g^−1^ in the 1 and 10 L cultures, but in the 250 L culture the yield decreased to 0.8 g galactaric acid [g d-galacturonate consumed]^−1^ after 72 h. The carbon balance (Fig. 3) indicated that all of the input carbon was accounted for in the output. This suggests that galactaric acid crystals may have been included with inadequately washed biomass in the dry weight determination, or that some d-galacturonate contributed to CO_2_ production through an unknown pathway. Up to around 7% galactaric acid was expected to be removed with the biomass, but is expected to be washed off during rinsing for the dry weight determination. However, when harvesting the 250 L culture we observed a decrease in galactaric acid concentration during the filtration process, which indicated that galactaric acid crystals were trapped as more biomass accumulated in the filter. The concentration of galactaric acid was determined after filtration. Large scale extraction of the biomass was not possible at this time, but it is clear that this would be needed to obtain the maximum galactaric acid output from a scaled up process.

In spite of the low yield at the 250 L scale, around 3.8 kg galactaric acid was produced and 2.8 kg was recovered by acid precipitation. Poor mixing and inadequate cooling in the vessels used for the crystallisation step will have contributed to the recovery of only 73% of the galactaric acid. This was adequate for supplying material for further chemical conversions, but highlights the need for further DSP development.

## Conclusions

The process of producing galactaric acid by bioconversion with *T. reesei* was demonstrated to be equally efficient using hydrolysed pectin as d-galacturonic acid as the substrate, and d-glucose present with the pectin did not inhibit the process. Scaling up to 250 L demonstrated good reproducibility with the smaller scale but there was a loss in yield at 250 L which indicated that both total biomass extraction and more efficient DSP would be needed for a large scale process. It would also be necessary to obtain higher production titres and production rates before a biotechnological process for producing galactaric acid would be considered for commercial production. However, the low solubility of galactaric acid [7] made it feasible to produce kilogram amounts of galactaric acid with the currently available strains so that assessment on purity requirements and further chemical transformations can proceed in parallel with further strain engineering. The 24-well plate batch process will be useful in screening new constructs, but cannot replace process optimisation in bioreactors.

## Methods

### Strains

*Trichoderma reesei* QM6a (VTT D-071262T, ATCC13631), M122 (RutC-30 *mus53*-), M1123 (M122 *pyr4*-) and VTT D-161646 were obtained from VTT’s strain collections. Spore suspensions were prepared by cultivating the fungus on potato-dextrose agar (BD, Sparks, Maryland, USA) for 5–7 days, after which the spores were harvested, suspended in a buffer containing 0.8% NaCl, 0.025% Tween-20 and 20% glycerol, filtered through cotton and stored at −80 °C. *Saccharomyces cerevisiae* strain H3488 (FY834, obtained from Jay C. Dunlap) was used to clone expression construct plasmids. *Escherichia coli* TOP10 (Invitrogen) was used for propagation of the plasmids.

### Expression vectors

Expression plasmids contained the 5′ and 3′ flanking regions of *T. reesei* d-galacturonic acid reductase (*gar1*, [18]), *T. reesei* promoter (*cbh1*, *cDNA1* or *pdc1*), *Agrobacterium tumefasciens* UDH gene, *Aspergillus trpC* terminator, *T. reesei pyr4* selection marker with a loop-out fragment for marker removal, and the pRS426 plasmid as vector backbone.

Plasmid pRS426 (ATCC77107), used in cloning expression construct plasmids in yeast, was obtained from Jay C. Dunlap. For cloning, pRS426 was digested with restriction enzymes *Eco*RI and *Xho*I. A plasmid containing *A. tumefasciens* d-galacturonic acid dehydrogenase (*udh* gene, GenBank no. BK006462.1) codon optimised for *Aspergillus niger*, [8] was digested with *Nco*I and *Sac*II to release the *udh* gene from the vector backbone. The 5′ and 3′ flank (1572 and 1500 bp) fragments of *gar1*, the three promoters (*cbh1, cDNA1* and *pdc1*) and 285 bp of the *gar1* 3′ flank as a direct repeat for looping out the selection marker were obtained by PCR using strain QM6a as template. The *Aspergillus trpC* terminator (773 bp) was obtained by PCR from a plasmid containing the element. PCR amplification was performed with a KAPA HiFi HotStart ReadyMix PCR kit (KAPABiosystems) using the primers listed in Table 4. The selection marker *pyr4* was obtained from an existing plasmid with *Not*I digestion. All PCRs and digestions were separated with agarose gel electrophoresis and correct fragments were extracted with a gel extraction kit (Qiagen).

**Table 4:** Primers used to generate fragments for cloning by PCR amplification

Product	Primer	Sequence	Product size (bp)
*gar1* 5′flank (tre22004)	MU01_gar1_5f_for	GGTAACGCCAGGGTTTTCCCAGTCACGACGGTTTAAACTTATATCCACCGTGTCCCAG	1610
	MU02_gar1_5f_rev	GACGCAGTTGTTTGAGCAAC	
*trpC* terminator (Aspergillus)	MU03_trpCt_for	GTGGATAACCCCATCTTCAAGCAGTCCTGAGATCCACTTAACGTTACTGAAATCA	803
	MU04_trpCt_rev	GAGTGGAGATGTGGAGTGGG	
*gar1* 3′direct repeat (tre22004)	MU05_gar1_3dr_for	TGTGTAAGCGCCCACTCCACATCTCCACTCGGCGCGCCTTGCATTGGTCAGAGCGGTA	361
	MU06_gar1_3dr_rev	CGAGAGCAGAGCAGCAGTAGTCGATGCTAGGCGGCCGCCCGACTTGGAGAAGCTCGTC	
*gar1* 3′flank (tre22004)	MU07_gar1_3f_for	CCAGCTGCGATTGATGTGTATCTTTGCATGGCGGCCGCTTGCATTGGTCAGAGCGGTA	1576
	MU08_gar1_3f_rev	AGCGGATAACAATTTCACACAGGAAACAGCGTTTAAACAAGCAGTGGATGACTTGCTG	
*cbh1* promoter (tre123989)	MU09_cbh1p_for	AGGTTAGTAGGTTGCTCAAACAACTGCGTCGGCCGGCCTGTGGCAACAAGAGGCCAGA	1646
	MU10_cbh1p_rev	GGCAGCGCCGGTGACGAGCAGGCGCTTCATGATGCGCAGTCCGCGGTTGA	
*cDNA1* promoter (tre123515)	MU11_cDNA1p_for	AGGTTAGTAGGTTGCTCAAACAACTGCGTCGGCCGGCCGAATTCGGTCTGAAGGACGT	1231
	MU12_cDNA1p_rev	GGCAGCGCCGGTGACGAGCAGGCGCTTCATGTTGAGAGAAGTTGTTGGATTGA	
*pdc1* promoter (tre121534)	MU13_pdc1p_for	AGGTTAGTAGGTTGCTCAAACAACTGCGTCGGCCGGCCAAAGGAGGGAGCATTCTTCG	1370
	MU14_pdc1p_rev	GGCAGCGCCGGTGACGAGCAGGCGCTTCATGATTGTGCTGTAGCTGCGCT	

The gene identifiers (tre-numbers) refer to Joint Genome Institute *T. reesei* assembly release version 2.0

The appropriate purified DNA fragments were transformed to *S. cerevisiae* FY834 using the method described in Gietz and Woods [19]. The plasmids obtained through homologous recombination reactions were isolated from *S. cerevisiae*, amplified in *E. coli* and checked by restriction enzyme digestions and sequencing.

### Strain generation

Expression plasmids (10 µg) were digested with *Mss*I, resulting in approximately 6 µg of released expression cassette from each. To generate 3′ single stranded overhangs and thus to improve transformation efficiency, digestion mixtures were further treated with T7 exonuclease [20] and used without further purification.

Strain M1123 was transformed with each of the three expression constructs and positive transformants were selected on minimal medium as described previously [21, 22]. Transformants were sub-cultured onto agar-solidified minimal medium containing 1 mL L^−1^ Triton X-100. The isolates were screened by PCR to verify correct integration at the *gar1* locus and a few correctly integrated transformants were purified as single spore isolates. Deletion of *gar1* was verified by PCR.

### Media

The low phosphate medium described by [7] was used for 24-well plate, flask and bioreactor cultivations, with d-galacturonic acid, pectin and lactose provided as carbon sources, as indicated. Medium for 24-well plate cultivations contained 15 g L^−1^ lactose with 10 g L^−1^ d-galacturonate or 10 g L^−1^ lactose with 20 g L^−1^ pectin (Sigma P1935, Table 2). Immediately before inoculation, 1 mL L^−1^ Pectinex Ultra SP-L (Novozymes) was added to pectin-containing medium. All 24-well plate medium also contained 100 mM PIPPS and 1 g L^−1^ spent grain extract, with the pH adjusted to 5.5.

Pre-cultures for bioreactors were grown in low phosphate medium with 20 g L^−1^ lactose and 1 g L^−1^ yeast extract.

Media for bioreactors contained 15 g L^−1^ lactose and 7.5 g L^−1^ Sigma pectin or 9 g L^−1^ lactose and 11 g L^−1^ Meridianstar Rapid set pectin (Table 2) in the batch phase, plus 3 g L^−1^ yeast extract. Filter sterilised Pectinex Ultra SP-L (1 mL L^−1^) was added to the reactors after sterilisation at 1 and 10 L scale, but unsterilized enzyme was added before sterilisation for the 250 L pilot. Feed for the fed-batch cultivations contained 1 g L^−1^ yeast extract and either 50 g L^−1^ Sigma pectin with 6 g L^−1^ lactose or 78 g L^−1^ Meridianstar pectin. Pectin in the feed was hydrolysed by the addition of 1.0 mL L^−1^ Pectinex Ultra SP-L with 0.1 mL L^−1^ Pectinex Smash (Novozymes) and incubation at ~45 °C, with agitation, prior to sterilisation. Hydrolysis was allowed to proceed until the viscosity had been reduced to allow adequate sterilisation. The d-galacturonic acid concentration was assessed by HPLC and if hydrolysis was not yet complete, additional sterile Pectinex Ultra SP-L was added after sterilisation to ensure the availability of d-galacturonic acid for the process.

### Culture conditions

Twenty-four-well plates (Whatman 10 mL round bottom Uniplate) contained 4 mL medium per well, with either 10 g L^−1^ d-galacturonate or 20 g L^−1^ pectin (Sigma), which was enzymatically hydrolysed simultaneously with the cultivation, as the substrate and lactose as the carbon source for growth. Wells were inoculated with approximately 10^5^ spores in 10 µL suspension (~2.5 × 10^4^ spores mL^−1^ final concentration). One well for each medium was not inoculated and used as a control. Three wells were inoculated with each new transformant (expressing *udh* under the *CBH1*, *PDC1* and *cDNA1* promoters) and one well each with M122 (negative control) and VTT D-161646. The plates were covered with adhesive, breathable rayon fibre film for culture plates (VWR) and incubated at 28 °C, 800 rpm (Infors HT Microtron, Switzerland), 85% relative humidity for up to 7 days. Samples (100 µL) were taken daily after 72 h using wide-mouth 200 µL pipette tips and either diluted and analysed immediately or stored at −20 °C. Sample size was minimised for the 24-well plates to ensure adequate culture volume for continued incubation.

Pre-cultures for bioreactor inocula were started in Erlenmeyer flasks (500–2000 mL containing 20% volume medium) inoculated with ~1 × 10^5^ spores mL^−1^ (final concentration) to provide a 10% v/v inoculum for 1, 10 or 32 L reactors. The 32 L reactor (New Brunswick 32 L BioFlo 510, Eppendorf) was used to provide inoculum for the 300 L reactor. Flasks were incubated at 30 °C with 200 rpm agitation for approximately 45 h before being transferred directly to bioreactors to provide an initial biomass of about 1 g L^−1^.

Bioreactor cultures were grown in Multifors (Infors HT, Switzerland, max. working volume 0.5 L), Biostat Q (Sartorius AG, Germany, 1 L max. working volume), Biostat C (Sartorius AG, Germany, 10 L max. working volume), BioFlo 510 (Eppendorf) or New Brunswick Scientific IF400 (Eppendorf, 350 L max. working volume) bioreactors. Cultures were maintained at 35 °C, with 500–900 rpm (0.5 L), 350–500 rpm (1 L), 400 rpm (10 L), 200 rpm (32 L) or 125–200 rpm (350 L reactor) agitation and approximately 0.5 volume gas (volume culture)^−1^ min^−1^ (vvm). Culture pH was kept constant at pH 4.0 by the addition of sterile 2–4 M NaOH or 1 M H_3_PO_4_. Gas concentration (CO_2_, O_2_, N_2_ and Ar) was analysed continuously using a Prima Pro Process mass spectrometer (Thermo Scientific, UK) calibrated with 3% CO_2_ in Ar, 5% CO_2_ with 0.99% Ar and 15% O_2_ in N_2_, 20% O_2_ plus 20% Ar in N_2_, and 0.04% ethanol in N_2_. Fed-batch cultures were provided feed at a constant rate after at least 5 g L^−1^ biomass had been produced. The start of feeding for the 1 L culture was delayed to allow complete utilisation of the lactose, but this appeared to reduce productivity and the feed for the 250 L culture (using the same substrate) was started with about 2 g L^−1^ lactose still present in the medium, as was the 10 L culture.

### Chemical analyses

The concentrations of d-glucose, lactose, d-galactose, d-galacturonic acid, and galactaric acid were determined by HPLC using a Fast Acid Analysis Column (100 × 7.8 mm, BioRad Laboratories, Hercules, CA) linked to an Aminex HPX-87H organic acid analysis column (300 × 7.8 mm, BioRad Laboratories) with 5 mM H_2_SO_4_ as eluent and a flow rate of 0.3 or 0.5 mL min^−1^. The column was maintained at 55 °C. Peaks were detected using a Waters 410 differential refractometer and a Waters 2487 dual wavelength UV (210 nm) detector.

Samples were diluted with eluent to give expected concentrations of galactaric acid between 1 and 3 g L^−1^ and heated at 100 °C for 1 h to solubilise crystals of galactaric acid prior to HPLC analysis. Samples from 24-well plates were diluted without separation of biomass and supernatant, heated at 100 °C for 0.5–1 h, then centrifuged at room temperature to remove biomass.

### Downstream processing

Culture broth was collected from the 1, 10 and 250 L cultures and the mycelium separated from the supernatant by filtration. At the 1 and 10 L scale, the mycelium was filtered through thick cleaning cloth under vacuum. The 250 L culture was warmed to 45 °C before biomass was separated from the liquid using Seitz K300 depth filter sheets (Pall Corporation) in a Seitz 40–30A4 filter press to obtain approximately 250 L filtrate and 20 kg wet biomass. The supernatant was transferred during filtration to 2 large vessels (165 and 85 L) which were cooled via cold jackets operated with water circulated from a chiller to give temperatures between 7 and 12 °C. One and 10 L volumes were stored at 4 °C. HCl was added to all culture supernatants to reduce the pH to between 1.5 and 2.2. Supernatants with precipitating galactaric acid crystals were mixed periodically by shaking (1 and 2 × 5 L) or stirring with a paddle (165 and 85 L) until it was convenient to collect the crystals of galactaric acid.

Galactaric acid crystals were collected by filtration through Whatman GF/A filter paper. For large volumes, the crystals were first allowed to settle to the bottom of the container and the clear liquid decanted to a clean vessel. The decanted liquid was expected to contain some galactaric acid and was returned to the cold to allow further crystal formation, precipitation and collection. The process was repeated twice for the 1 L culture supernatant and four times for the 10 and 250 L volumes.

### Biomass determination

Mycelia were collected by filtration through Whatman GF/B filters under vacuum and washed twice with an equal or greater volume of distilled H_2_O. Mycelia were dried to a constant weight at 105 °C.
